# Recombinant Spider Silk Enhances Engineered Cartilage Formation

**DOI:** 10.3390/jfb17050252

**Published:** 2026-05-19

**Authors:** Hongji Zhang, Xinyu Huang, Jinwen Zhang, Fengjie Zhang, Fei Sun, Chao Wan

**Affiliations:** 1 Key Laboratory of Regenerative Medicine, Ministry of Education, School of Biomedical Sciences, Faculty of Medicine, The Chinese University of Hong Kong, Hong Kong SAR, China; zhan1754@link.cuhk.edu.hk (H.Z.); 1155203741@link.cuhk.edu.hk (J.Z.); b123228@cuhk.edu.hk (F.Z.); 2 Center for Neuromusculoskeletal Restorative Medicine, Hong Kong Science Park, Hong Kong SAR, China; 3 Key Laboratory of Regeneration Medicine (Shenzhen Base), Ministry of Education, School of Biomedical Sciences Core Laboratory, Shenzhen Research Institute, The Chinese University of Hong Kong, Shenzhen 518172, China; 4 Oriental Regeneration Ltd., Hong Kong SAR, China; 5 Department of Chemical and Biological Engineering, School of Engineering, The Hong Kong University of Science and Technology, Hong Kong SAR, China; xhuangbn@connect.ust.hk

**Keywords:** recombinant spider silk (RSS), protein hydrogel, hydrogel composite, biomaterials, cartilage tissue engineering

## Abstract

Articular cartilage is characterized by its avascular, aneural, and alymphatic nature, which confers a limited intrinsic capacity for self-repair. Current regenerative strategies primarily focus on alleviating pain, mitigating symptoms, and restoring joint function. However, their long-term efficacy remains uncertain. Cartilage tissue engineering has emerged as a promising alternative to conventional therapies, offering innovative solutions for articular cartilage regeneration. Central to this approach is the development of functional biomaterials capable of supporting chondrogenic cell adhesion, proliferation, and differentiation, thereby facilitating effective cartilage repair. In this study, we introduce a novel protein-based recombinant spider silk (RSS) as a potential biomaterial for modulating chondrocyte behavior and enabling engineered cartilage formation both in vitro and in vivo. RSS was generated through molecular cloning and processed into silk fibers using biomimetic spinning and acidic coagulation techniques. In micromass cultures of murine chondrocytes, RSS significantly promoted cell aggregation, resulting in increased cell density. Alcian blue and Oil Red O staining demonstrated that RSS-treated cultures produced abundant glycosaminoglycans, a hallmark of chondrogenic activity, while exhibiting minimal lipid accumulation. These findings suggest that RSS supports chondrogenic differentiation and suppresses adipogenic lineage commitment. Real-time PCR analysis revealed upregulation of the chondrogenesis-related gene Sox9 and downregulation of the adipogenic marker PPARγ and the hypertrophic marker Runx2 in RSS-treated micromass cultures. RNA sequencing further corroborated these observations, underscoring the role of RSS in modulating extracellular matrix (ECM) remodeling in chondrocytes. In a subcutaneous transplantation model using severe combined immunodeficiency (SCID) mice, chondrocytes encapsulated in three-dimensional hydrogel scaffolds containing RSS exhibited significantly enhanced ECM accumulation compared to RSS-free controls, indicating that RSS supports the maintenance of the chondrocyte phenotype and promotes cartilage formation in vivo, and underscoring its promising potential as a component of hydrogel composite systems. These findings highlight the potential of RSS as a functional biomaterial to preserve chondrocyte functionality and advance engineered cartilage formation, presenting a promising avenue for cartilage tissue engineering and regeneration.

## 1. Introduction

Cartilage is a resilient and supportive connective tissue characterized by an extensive extracellular matrix (ECM) and chondrocytes housed within lacunae [1]. Based on differences in ECM composition, it is categorized into three subtypes: hyaline cartilage, elastic cartilage, and fibrocartilage [2]. Among these, hyaline cartilage is most prevalent at the sternal ends of the ribs and on articular surfaces of bones, where it is referred to as articular cartilage. The primary functions of articular cartilage include facilitating near-frictionless joint movement, absorbing and distributing mechanical loads, and protecting the underlying subchondral bone [3]. Nevertheless, the continuous mechanical stress imposed on articular cartilage renders it vulnerable to wear, tears, and sports-related injuries [4]. Due to its avascular nature, lack of innervation, and low cellular density, articular cartilage exhibits a very limited intrinsic capacity for self-repair and regeneration [5]. In the absence of effective intervention, articular cartilage damage often leads to progressive tissue degradation, chronic joint pain, and functional impairment, ultimately culminating in osteoarthritis (OA) [6]. OA is one of the most prevalent degenerative joint diseases and is associated with a high rate of disability. Its incidence continues to rise with the aging population and increasing rates of obesity, making OA an escalating global health concern. Current clinical estimates suggest that approximately 595 million people worldwide are affected by OA. Despite its widespread impact, the underlying pathogenesis of OA remains incompletely understood, and existing therapeutic strategies are insufficient to fully meet clinical needs [7,8].

Current therapeutic strategies for managing articular cartilage damage or OA primarily aim to alleviate pain and improve joint function. However, they fail to restore native hyaline cartilage with its characteristic structural and mechanical properties, rendering these interventions only partially effective [9]. Microfracture surgery, for instance, involves creating small perforations in the subchondral bone to recruit bone marrow-derived mesenchymal stem cells (MSCs) into the defect region. Although these cells undergo proliferation and chondrogenic differentiation, the resultant repair tissue is typically fibrocartilage, which lacks the biomechanical resilience of hyaline cartilage [10]. Autologous cartilage transplantation addresses focal defects by harvesting cartilage from non-load-bearing regions and implanting it into the injured area. Clinical evidence indicates satisfactory graft integration and defect filling, with restoration of biological function. Nevertheless, this approach is constrained by limited donor tissue availability, potential donor site morbidity, and biomechanical discrepancies between donor and recipient sites [11]. Allogeneic cartilage grafting circumvents donor site limitations but introduces the risk of immune rejection, thereby restricting its widespread adoption [12]. To overcome the limitations of current OA therapies, the development of improved and innovative treatment strategies is imperative. Tissue engineering, which integrates principles from the life sciences and engineering to create biological substitutes capable of restoring or replacing damaged tissues, represents a promising approach to addressing these challenges [13]. Various advanced fabrication techniques—including electrospun hydrogel fiber [14,15,16], injectable hydrogels [17,18,19], and three-dimensional (3D) bioprinted hydrogels [20,21,22]—have been extensively utilized in cartilage tissue engineering applications. The successful application of this strategy requires a comprehensive understanding of the physiological properties and pathological alterations characteristic of cartilage tissue, as well as the rational design and optimization of functional biomaterials to effectively promote cartilage regeneration [23]. Spidroin, a naturally derived protein, has emerged as a versatile biomaterial with broad applications in tissue engineering [24]. Its tunable biomechanical properties facilitate favorable interactions between the biomaterial and surrounding cells [25]. Key characteristics of the spidroin-based biomaterials, particularly in hydrogel form—including mechanical performance, swelling behavior, and degradation rate—are governed primarily by the content of silk II (β-sheet) structures and can be modulated through various modification strategies, underscoring the material’s significant plasticity [26]. In comparison to other natural biomaterials such as collagen, spidroin exhibits superior mechanical strength in vivo [27]. Moreover, spidroin-based substrates provide a supportive microenvironment for cell proliferation, differentiation, and ECM remodeling. Spidroin-based biomaterials are not limited to fibrous architectures; recombinant spider silk proteins have also been engineered into physically and chemically crosslinked hydrogels with tunable mechanical and swelling properties. These silk-derived hydrogel systems exhibit high water content, ECM-mimetic properties, and favorable cell–material interactions, positioning spidroin as a versatile platform for gel-based tissue engineering applications [28]. Previous studies have demonstrated that spidroin components enhance the expression of cartilage matrix constituents, highlighting their potential in promoting hyaline cartilage repair [29]. Nevertheless, the development of functionalized spidroin derived biomaterials, including fiber, foam and hydrogel, capable of supporting chondrogenic cell adhesion, proliferation, and differentiation remains a critical objective for advancing cartilage tissue engineering.

**Figure 1:**
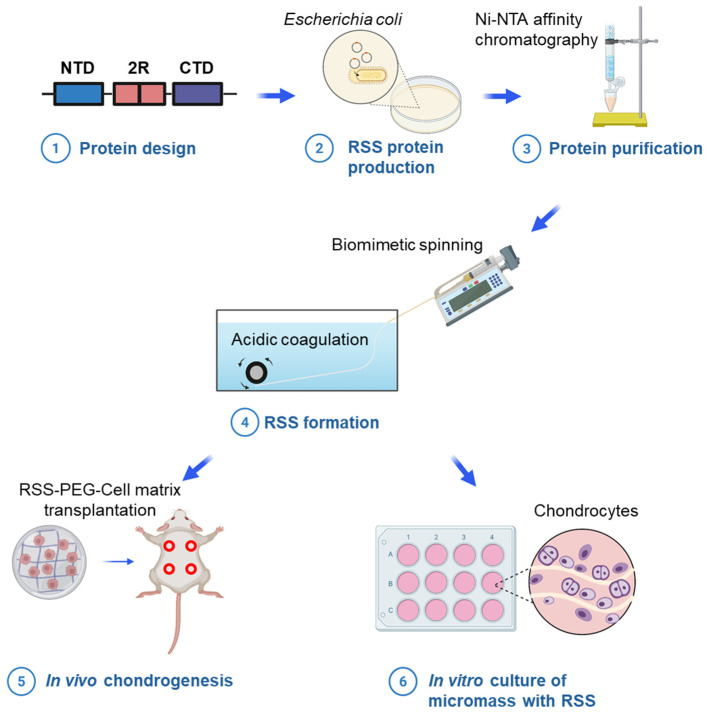
Schematic illustrating the generation of RSS through molecular cloning and biomimetic spinning, followed by evaluation of its biological functionality in vitro and in vivo. The diagram was created using BioRender.com.

Building upon our previous work, in which we developed one-dimensional recombinant spider silk (RSS) using genetically encoded click chemistry for various biomedical applications [30], the present study aims to further investigate the biological effects of RSS in promoting chondrogenic differentiation while inhibiting adipogenic commitment in murine chondrocytes in vitro, to elucidate the molecular mechanisms underlying RSS-induced chondrogenesis through transcriptomic analysis, and to evaluate the ability of RSS, when incorporated into polyethylene glycol (PEG) hydrogel scaffolds, to maintain the chondrocyte phenotype and enhance engineered cartilage formation in vivo (Figure 1). The RSS fabrication process began with molecular cloning to produce spidroin proteins, followed by purification via Ni-NTA affinity chromatography to obtain the spidroin dope. To mimic the natural spinning process of the major ampullate gland in spiders, the protein solution was processed into silk fibers and coagulated in an acidic buffer (pH 5.0). The resulting RSS exhibited a notable capacity to induce cell aggregation and support chondrogenic differentiation while concurrently suppressing adipogenic lineage commitment. In vitro, chondrocyte micromass cultures seeded onto RSS showed increased glycosaminoglycan (GAG) accumulation, enhanced cell aggregation, and minimal lipid droplet formation under chondrogenic induction conditions. Real-time PCR analysis confirmed that RSS upregulated the expression of chondrogenic marker genes and downregulated adipogenic marker genes. Furthermore, RNA sequencing revealed that RSS modulated the expression of genes involved in ECM organization, collagen degradation, and ECM remodeling, underscoring its regulatory role in these critical processes. In vivo, chondrocytes encapsulated within 3D hydrogel scaffolds containing RSS exhibited significantly greater ECM accumulation than those in RSS-free controls, indicating that RSS helps preserve the functional phenotype of encapsulated chondrocytes and supports robust cartilage formation. Compared with existing biomaterials used in cartilage tissue engineering, RSS offers several distinct advantages. Unlike naturally derived spider silk, RSS enables scalable production with high purity and minimal batch variability through controlled recombinant expression systems. Compared to natural polymers such as collagen or hyaluronic acid, RSS exhibits enhanced mechanical stability and resistance to enzymatic degradation. In contrast to synthetic polymers such as PEG or Poly Lactic-co-Glycolic Acid (PLGA), which lack intrinsic bioactivity, RSS demonstrates inherent chondroinductive properties without requiring additional functionalization. Furthermore, RSS possesses unique dual functionality by promoting chondrogenesis while suppressing adipogenesis, which is particularly relevant in the context of OA [31]. The genetically programmable nature of RSS also enables further functionalization and structural tuning, providing a versatile platform for advanced tissue engineering applications. Collectively, these findings demonstrate that RSS holds strong promise as a platform for constructing and maintaining engineered cartilage, offering a novel biomaterial and strategy to advance cartilage tissue engineering and regeneration.

## 2. Materials and Methods

### 2.1. Generation and Characterization of RSS Biomaterials

RSS was genetically engineered and expressed following the previously established method [30]. Briefly, fusion genes were designed and constructed for cloning using synthetic oligonucleotides and then amplified by PCR. The expression vector pET22b (+) was employed for the expression of the fusion genes. Plasmid constructs were introduced into the Escherichia coli strain DH5α for plasmid amplification and molecular cloning. Subsequently, the amplified plasmids were transformed into the Escherichia coli strain BL21 for protein expression. The resulting proteins were purified under denaturing conditions using Ni-NTA resin (Cytiva, Marlborough, MA, USA), which specifically binds to the 6× His fusion tag at the C terminus of the proteins. The purified proteins were characterized using SDS/PAGE and Coomassie Brilliant Blue staining. A 1 mL syringe with a Luer-Lok tip was filled with 250 mg mL^−1^ spinning spidroin dope and connected to a 34 G stainless steel blunt tip needle with an inner diameter of 0.06 mm. The concentrated spinning dope, a type of protein gel, was extruded into an acidic coagulation bath containing 500 mM sodium acetate (NaAc) and 250 mM NaCl (pH 5.0) at a smooth flow rate of 1.2 mL h^−1^ using a syringe pump (Pump 11 Pico Plus Elite, Harvard Apparatus, Holliston, MA, USA). Silk threads were collected by a submerged roller and kept in the spinning buffer for at least 48 h before use. Subsequently the fabricated silk fiber was treated with UV light overnight, and finally stored in 1× phosphate-buffered saline (PBS) at 4 °C. To assess the maintenance of silk fiber morphology after immersion in PBS, wet RSS samples were imaged using a 2× objective lens on a ZEISS stereomicroscope (Oberkochen, Germany). Porosity and surface features were further analyzed with a JEOL-7800F field emission scanning electron microscope (Akishima, Japan) operated at an acceleration voltage of 10.0 kV. SEM analysis revealed an average fiber diameter of approximately 140 μm. Prior to imaging, the scaffolds were freeze-dried and sputter-coated with a thin layer of platinum (SPI). Micrographs were obtained using a secondary electron detector, and element distribution across the RSS thread cross-section was analyzed using energy dispersive X-ray spectroscopy (EDX).

### 2.2. Murine Primary Chondrocytes Isolation and Culture

Murine chondrocytes were isolated and cultured according to established protocols [32]. In brief, primary chondrocytes were obtained from the ribs of C57BL/6 newborn mice euthanized by Isoflurane anesthesia. The ribs were digested with 0.075% collagenase I and 0.3% collagenase D, and the resulting cell suspension was filtered through a 70-µm strainer to remove larger tissue fragments. After centrifugation, the isolated chondrocytes were plated in a 150 mm Petri dish containing α-MEM supplemented with 1% penicillin/streptomycin sulfate, 1% L-glutamine, 10% fetal bovine serum, and incubated at 37 °C in a 5% CO_2_ humidified environment. On the second day, non-adherent cells were removed, and the cultures were gently rinsed twice with PBS before being replenished with fresh medium. The adherent cells were maintained with medium changes every two days until they reached confluence. First-passage cells or second-passage cells were used for subsequent experiments.

### 2.3. Chondrocyte Micromass Culture with RSS

Before use in cell culture, the fabricated spider silk was further sterilized by rinsing it with 70% vol/vol ethanol in deionized water. After washing the 2 mm spider silk fibers in PBS to remove any residual ethanol, the material was cut into fiber fragments and placed into individual wells of a 24-well plate. Confluent monolayer cultures of primary chondrocytes from mice were then released using trypsin-EDTA and resuspended in serum-free medium at a density of 1 × 10^5^ cells/10 μL. Micromasses were formed by pipetting 10 μL of the cell suspension into each well of the 24-well plate. After a 3 h attachment period without medium, growth medium (α-MEM with 10% FBS, 1% P/S, and 1% L-glutamine) was gently added, and the micromass culture was allowed to rest for an additional 48 h at 37 °C in a humidified 5% CO_2_ atmosphere. The medium was replaced every 2 days, with the day of micromass plating designated as day 0. On day 3 of the culture, some micromasses were harvested for Alcian blue matrix staining, Oil Red O staining, and others for quantitative reverse transcription-polymerase chain reaction (qRT-PCR) analysis to examine the expression of marker genes for chondrogenesis and adipogenesis: type II collagen (Col II), type I collagen (Col I), type X collagen (Col X), sex-determining region Y-box 9 (SOX9), peroxisome proliferator-activated receptor gamma (PPAR-γ), and runt-related transcription factor 2 (RUNX2).

### 2.4. Alcian Blue Staining and Oil Red O Staining

For chondrogenesis assay, the micromasses were cultured for 3–4 days and rinsed twice with PBS, then fixed for 20 min with 0.5 mL of 4% paraformaldehyde (PFA) at room temperature. The micromasses were then washed with sterile distilled, deionized water and stained with 0.5 mL of 1% (*w*/*v*) Alcian blue (Sigma, St. Louis, MO, USA) in glacial acetic acid (3%) (*v*/*v*), which was added to each well for 30 min or 1 h at room temperature. After two washes with 70% (*v*/*v*) ethanol and three washes with sterile distilled, deionized water, photomicrographs of the stained cell mass were obtained using a Ti2-E microscope (Nikon, Tokyo, Japan). The ECM proteoglycan was stained blue. For adipogenesis assay, micromasses were collected and fixed as above, and then washed with PBS to remove any residual fixative. The samples were then rinsed with 60% isopropanol for 5 min to enhance the binding efficiency of the subsequent reaction. They were then incubated with filtered 0.3% Oil Red O (Sigma, St. Louis, USA) working solution in isopropanol for 15 min, followed by a thorough rinse with deionized water. The positive staining areas were imaged using a Nikon Ti2-E microscope. The lipid droplet was stained red. The quantitative analysis was evaluated and analyzed using ImageJ 1.53 software.

### 2.5. Real-Time PCR

Total RNA was extracted and purified using a commercially available kit from Zymo (Orange, CA, USA), following the manufacturer’s guidelines. The concentration and purity of the isolated RNA were assessed using the NanoDrop 2000 (Thermo Fisher, Waltham, MA, USA). Complementary DNA (cDNA) was synthesized by reverse transcribing 0.5 µg of total RNA with the PrimeScript RT Master Mix system (Takara, Otsu, Japan), according to the manufacturer’s protocol and using oligo dT primers. Quantitative real-time PCR was performed using the TB Green Premix Ex Taq II (Takara, Otsu, Japan) in ABI QuantStudio 7 System (Applied Biosystems). Each reaction was performed in triplicates under the following parameters: 95 °C for 30 s and 40 cycles of 95 °C for 5 s and 60 °C for 34 s. β-actin was used as an internal control. Primer sequences are shown in Table 1.

### 2.6. Transcriptomic Analysis (mRNA-Seq)

Chondrocyte micromass samples cocultured for 7 days on RSS, referred to as the silk group, were collected for analysis. Total RNA was extracted from these samples using TRIzol reagent, and RNA libraries were constructed before undergoing high-throughput sequencing (Novogene Co., Ltd., Beijing, China). Differentially expressed genes (DEGs) were identified using DESeq 2 software, with significance criteria set at a *p*-value below 0.05 and a fold change exceeding 2 or below 0.5. Furthermore, Gene Ontology (GO) enrichment analysis was performed for comprehensive bioinformatics analysis. 

### 2.7. Subcutaneous Transplantation In Vivo of Silk Loaded PEG Constructs

#### 2.7.1. Preparation of PEG Bioscaffolds with Silk for In Vivo Transplantation

PEG bioscaffolds were developed as vectors for evaluating the functionality of spider silk in cartilage repair in vivo, and they were categorized into three groups: (i) acellular constructs with silk, (ii) chondrocyte-laden constructs with silk, and (iii) chondrocyte-laden constructs without silk. To create the PEG bioscaffolds, 0.04 g of 4-arm PEG-VS (Jenkem) powder was dissolved in 1 mL of PBS. For RSS-containing groups, approximately 2 mg of sterilized, fragmented RSS fibers (1–2 mm in length) were physically mixed with 1 mL of 4-arm PEG-VS solution prior to crosslinking, yielding a final silk-to-polymer weight ratio of approximately 1:20. For cell encapsulation, incubated chondrocytes were digested with 0.25% Trypsin–EDTA, resuspended at a density of 4 × 10^7^ cells/mL, and thoroughly mixed with the PEG solution via pipetting before being transferred into sterilized syringe molds. The resulting PEG–cell mixtures were cross-linked using 2,2′-(Ethylenedioxy)diethanethiol (Sigma, St. Louis, USA), which provided an equimolar concentration of thiol groups to react with the vinyl sulfone groups from PEG-VS for 30 min at 37 °C, and then washed with PBS. The gelated blocks were incubated in fresh culture medium at 37 °C and 5% CO_2_ overnight and implanted the following day.

#### 2.7.2. Transplantation of Bioscaffolds in the SCID Mice

The study involved implanting prepared PEG blocks from three treatment groups into SCID mice. The blocks were first washed with serum-free media and kept in a 12-well plate during surgery. Before implantation, the scaffolds were blotted dry after a single PBS wash. A total of three SCID mice were used. In each mouse, four subcutaneous implants were placed, with two blocks from each of the three experimental groups—acellular control, PEG-only (chondrocyte-laden), and PEG–RSS (chondrocyte-laden)—following a randomized distribution manner. The design ensured a balanced allocation across groups, resulting in four biological replicates per treatment condition. To alleviate pain, the animals were anesthetized with a mixture of isoflurane, xylazine, and ketamine before surgery. Anesthesia was maintained during the procedure with a mild inhalation of isoflurane. A total of four PEG constructs per treatment group were implanted into one mouse’s subcutaneous area, with two blocks on each side in a randomized manner. Incisions on the animals’ backs were closed using sterile metal surgical sutures, and the blocks were implanted sequentially. Following surgery, the mice received buprenorphine injections once daily for two days to further minimize discomfort. They were temporarily housed in temperature-controlled, ventilated cages set at 27 °C. After full recovery, the animals were housed in ventilated cages and monitored continuously.

### 2.8. Histological and Immunohistochemistry Analysis

The harvested samples from the SCID mice were fixed with 4% PFA solution for 12 h at 4 °C, filtrated with O.C.T. Compound (Sakura Finetek, Torrance, CA, USA) for 5 days and solidified by liquid nitrogen, then cryo-sectioned on a cryotome (NX70, Epredia, Kalamazoo, MI, USA). Sections with a thickness of 10 µm were collected on Polysine Adhesion Microscope Slides (Epredia, Kalamazoo, USA), and stained with Alcian blue, Safranin O to visualize and evaluate the ECM production. For immunohistochemical staining, Mouse and Rabbit Specific HRP/DAB (ABC) Detection IHC Kit (Abcam, Cambridge, UK) was used to perform following manufacturer’s instruction. Sections were blocked with 3% (*v*/*v*) hydrogen peroxide and bovine serum albumin (BSA) and incubated with the primary antibodies of rabbit polyclonal anti-Collagen type II (Col II) (Abcam, 1:100) and anti-Aggrecan (Millipore, St. Louis, MO, USA, 1:100). Adjacent sections were incubated with IgG as negative controls. All the images were analyzed and quantified by ImageJ 1.53 software.

### 2.9. Statistical Analysis

All statistical analyses were conducted using GraphPad Prism 9.0. Data are presented as the mean ± SD and visualized with the same software. Group comparisons between groups were performed using Student’s t-test, with statistical significance defined as a *p*-value less than 0.05.

## 3. Results and Discussion

### 3.1. Production of RSS

To enable scalable production of RSS by mimicking the natural spinning process of the major ampullate gland, a biomimetic spinning approach was employed. The setup consisted of a microinjection syringe pump loaded with protein spinning dope and connected to a 34 G stainless steel blunt-tip needle, through which continuous silk threads were extruded into an acidic coagulation bath (pH 5.0). This pH condition was selected based on prior reports demonstrating its efficacy in promoting the conformational transition of soluble spidroins into insoluble silk fibers [33]. A key advantage of this spinning system lies in its reliance on standardized industrial components, which simplifies fabrication compared to microfluidic-based or other complex approaches. The resulting silk threads exhibited smooth surfaces, uniform cross-sections, and remarkable stability—even under harsh conditions such as immersion in 0.1 M NaOH or exposure to 2.5% trypsin at 37 °C overnight. An overview of RSS fabrication and its subsequent evaluation in chondrogenesis is illustrated in Figure 1.

### 3.2. RSS Maintains Fiber Morphology Following Fabrication

**Figure 2:**
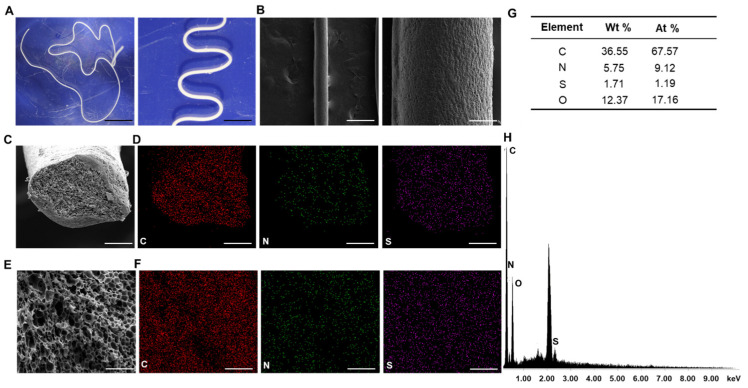
**Characterization of RSS.** (**A**) Dissection microscope image of hydrated RSS fibers. (**B**) SEM image of freeze-dried RSS showing surface morphology (top view). (**C**,**E**) SEM images of RSS cross-sections. (**D**,**F**) EDX elemental maps of RSS cross-sections. (**G**) EDX quantitative analysis showing atomic and weight percentages of each element across the entire cross-section. (**H**) EDX spectrum showing characteristic peaks of elements present in RSS. Scale bars: (**A**) 2 mm and 1 mm; (**B**) 250 μm and 50 μm; (**C**,**D**) 50 μm; (**E**,**F**) 10 μm. C, carbon; N, nitrogen; O, oxygen; S, sulfur.

During the biomimetic spinning process, RSS dope was exposed to shear forces along the syringe channel before being extruded into the acid coagulation bath. Optical microscopy reveals that after immersion in the coagulation buffer and PBS, RSS retains a uniform fiber morphology, demonstrating its good stability (Figure 2A). To examine the surface morphology of RSS, SEM analysis was conducted, revealing that the surface of the as-spun RSS dragline fiber exhibits fibril and network-like patterns (Figure 2B). This fibrillar structure has been observed in both natural spider dragline silk and recombinant dragline silk [34,35]. Additionally, the fabricated RSS fibers display noticeable cracks and granules on the fiber surface (Figure 2B), along with numerous irregular voids and pores in the cross-section (Figure 2C–E).

EDX analysis confirmed the presence of amino acids within the spun fiber (Figure 2H). The intense peak at approximately 2.1 keV corresponds to the platinum (Pt) Mα emission line (2.051 keV) originating from the Pt sputter-coating applied for SEM imaging. As anticipated, a small amount of sulfur was detected, originating from cystine and methionine, which are enriched in MaSp1 and MiSp, the main components of RSS protein. Additionally, nitrogen, a key element in the amino groups of amino acids, was observed, further validating the characteristics of RSS. Elemental mapping of sulfur and nitrogen, shown in Figure 2D–F, revealed a heterogeneous distribution across the entire cross-section of the thread.

### 3.3. RSS Promotes Chondrogenic Differentiation While Inhibiting Adipogenic Differentiation of Chondrocytes

Following the fabrication of the material, the effect of RSS on chondrocyte differentiation was evaluated. Chondrocytes were isolated from newborn mice, and micromass cultures were established by pipetting 20 µL of high-density (2 × 10^5^) cell suspension onto the silk material and incubating cells with chondrogenic medium for 7 days. For the control group, micromass were cultured in the 24-well plates without RSS.

**Figure 3:**
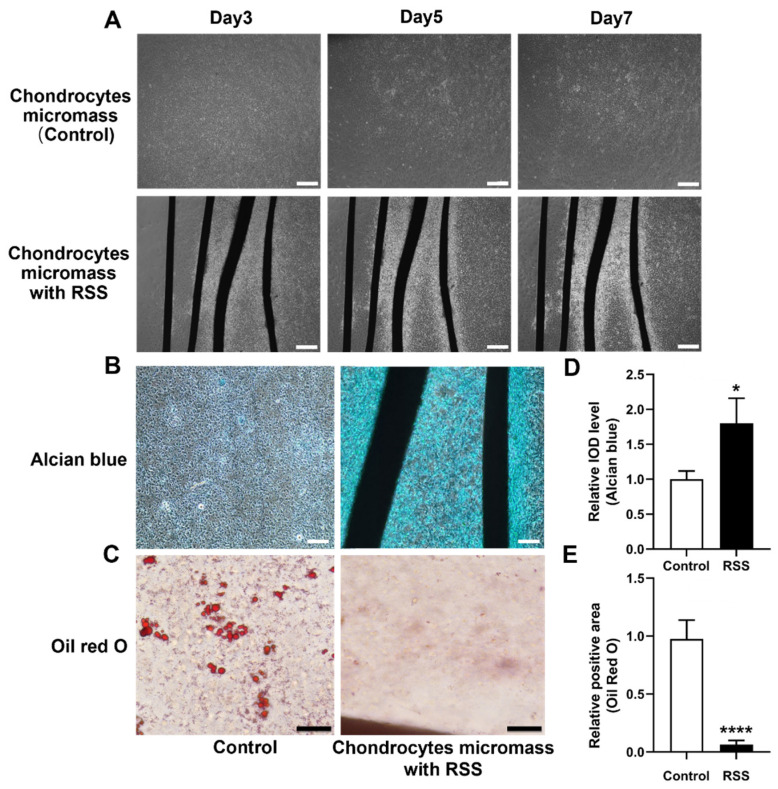
**RSS enhances chondrocyte aggregation and chondrogenesis while suppressing adipogenesis.** (**A**) Phase-contrast images of micromass cultures with and without RSS treatment. Scale bar: 400 μm. (**B**) Representative images of Alcian blue staining indicating glycosaminoglycan deposition in micromass cultures with and without RSS. Scale bar: 200 μm. (**C**) Representative images of Oil Red O staining showing lipid droplet formation in micromass cultures with and without RSS. Scale bar: 50 μm. (**D**) Quantitative analysis of integrated optical density (IOD) for Alcian blue staining. * *p* < 0.05, *n* = 3. (**E**) Quantification of Oil Red O–positive area. **** *p* < 0.0001, *n* = 3.

Using phase-contrast microscopy, it was observed that in the control group, cell distribution remained more homogeneous over 3, 5, and 7 days (Figure 3A). In contrast, in the RSS co-incubation group, cell density was significantly higher near the silk material, and the cells showed a tendency to align and grow along the material at all time points. This suggests that RSS facilitates cell aggregation, a critical factor for the phenotypic expression of chondrocytes.

On day 7, GAG content in the micromass culture from each group, a key marker of chondrogenesis, was assessed using Alcian Blue staining (Figure 3B). The results showed that micromass with RSS groups exhibited significantly higher staining intensity compared to the control groups, indicating more GAG content in the ECM (Figure 3C). These data suggest that RSS plays a vital role in promoting and accelerating chondrogenic differentiation. Meanwhile, Oil red O staining clearly showed that fewer lipid droplets were formed in micromass with RSS groups compared to the control groups (Figure 3D), suggesting reduced lipid production. Quantitative analysis confirmed that RSS significantly limited lipid accumulation in vitro (Figure 3E). These findings highlight RSS’s potential to inhibit adipogenic differentiation of chondrocytes, a process critical for cartilage development and regeneration.

### 3.4. RSS Upregulates Chondrogenesis-Related Marker Genes and Downregulates Adipogenesis-Associated Marker Genes

**Figure 4:**
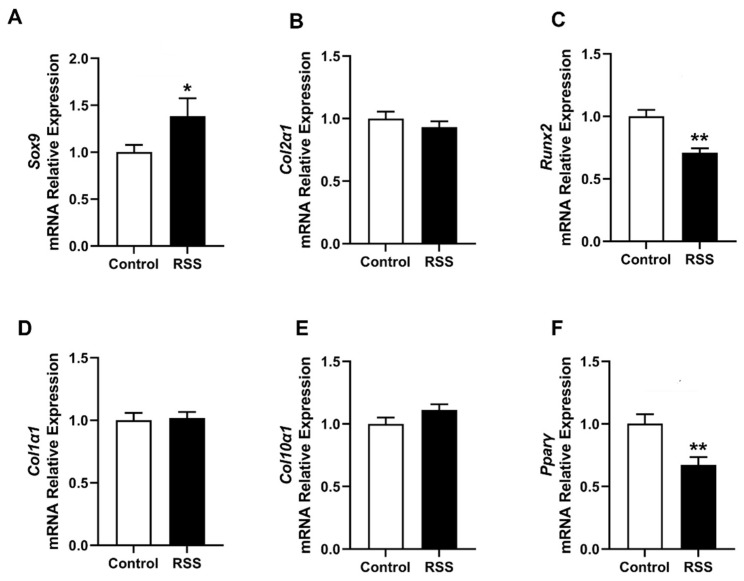
**Quantitative real-time PCR analysis of chondrogenesis-related and adipogenesis-associated gene expression in micromass cultures with and without RSS treatment.** Relative mRNA expression levels of (**A**) Sox9, (**B**) Col2α1, (**C**) Runx2, (**D**) Col1α1, (**E**) Col10α1, and (**F**) PPARγ were measured. The mRNA levels were normalized to β-actin levels. * *p* < 0.05, ** *p* < 0.01, *n* = 3.

To assess the effects of RSS on the chondrocyte phenotype in micromass cultures, we further analyzed the gene expression of cartilage markers, including Sox9 and Col2α1. The results showed that the addition of RSS significantly increased Sox9 expression, a key regulator of chondrogenesis, while having no significant impact on the expression of Col2α1, another typical cartilage marker (Figure 4A,B). These findings are consistent with other studies on silk fibroin-based designs [36], suggesting that RSS initiates chondrogenesis at the molecular level, though a longer incubation period may be required for significant upregulation of Col2α1.

For articular cartilage tissue engineering, promoting the desirable hyaline cartilage phenotype while minimizing undesirable fibrocartilage or hypertrophic phenotypes is critical. These phenotypes are characterized by the markers Col1α1, Runx2, and Col10α1 [37,38,39]. Compared to the control group, chondrocytes co-incubated with RSS exhibited significantly lower Runx2 expression and unchanged expression levels of Col1α1 and Col10α1 (Figure 4C–E). The observed decrease in RUNX2 expression in our study may indicate suppression of hypertrophic signaling, whereas the unchanged *Col10α1* expression could reflect compensatory regulatory mechanisms, temporal differences in gene expression, or the influence of the biomaterial microenvironment [40,41]. Such asynchronous regulation of hypertrophic markers has been previously reported in biomaterial-based chondrogenic differentiation systems, particularly during early-stage or in vitro conditions [42]. We acknowledge that protein-level confirmation of RUNX2 and Col10a1 is necessary. Future studies using RSS-based constructs will include immunoblotting or immunofluorescence for both markers to further determine the alterations of those markers at protein level.

Additionally, we examined the effects of RSS on PPARγ, a key marker of adipogenic differentiation [43]. Gene expression analysis revealed that the co-incubation of RSS with chondrocytes significantly downregulated PPARγ activity (Figure 4F). This suggests that the interaction between RSS and chondrocytes limits the tendency of chondrocytes to undergo adipogenic differentiation, further supporting RSS’s potential role in maintaining and enhancing the chondrogenic phenotype.

### 3.5. Transcriptomic Analysis of Chondrocyte Incubated with RSS

**Figure 5:**
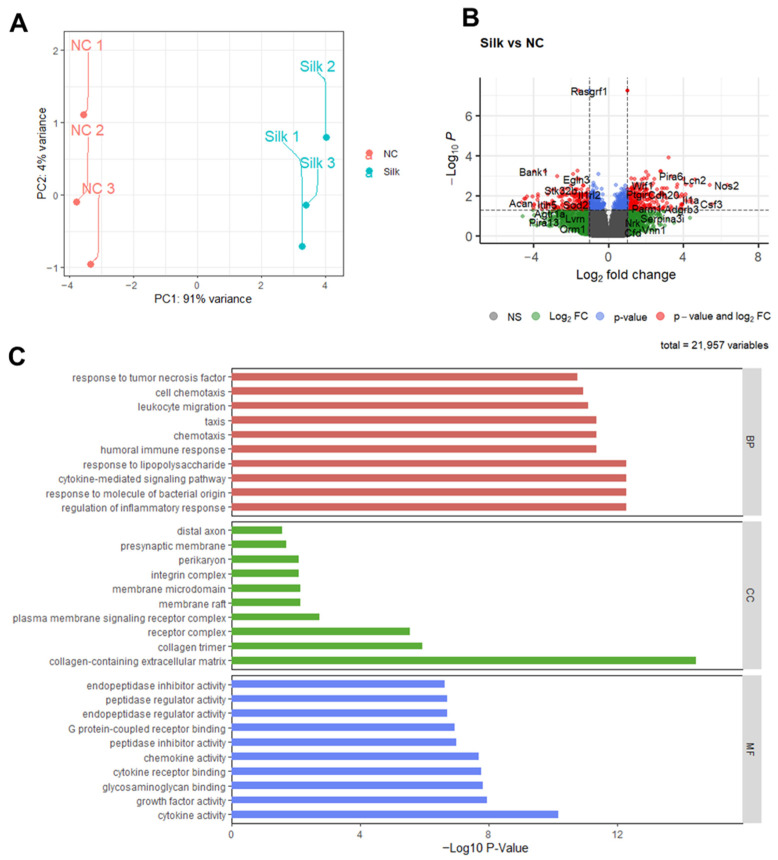
**Transcriptomic profiling of chondrocyte micromass cultures in response to RSS treatment using bulk RNA-seq.** (**A**) Principal component analysis (PCA) of differentially expressed genes (DEGs) in the control group and RSS group. (**B**) Volcano plot depicting DEGs in micromass cultures following 3 days of RSS treatment compared to control conditions. Genes with a minimum 2-fold change (adjusted *p* < 0.05) are highlighted. Red, significantly upregulated genes; blue, significantly downregulated genes; green, non-significant DEGs. (**C**) Gene Ontology enrichment analysis of DEGs identified in RSS-treated chondrocytes encompassing the Biological Process (BP), Cellular Component (CC), and Molecular Function (MF) categories.

To further explore the regulation of genes by RSS in chondrocytes at the transcriptional level, mRNA sequencing (mRNA-seq) analysis was performed. Principal component analysis (PCA) was used to compare gene expression patterns between the control and RSS-treated groups (Figure 5A). In the PCA plots, samples from the same group clustered closely together, indicating high reproducibility, while the separation between the groups highlighted significant differences in gene expression.

**Figure 6:**
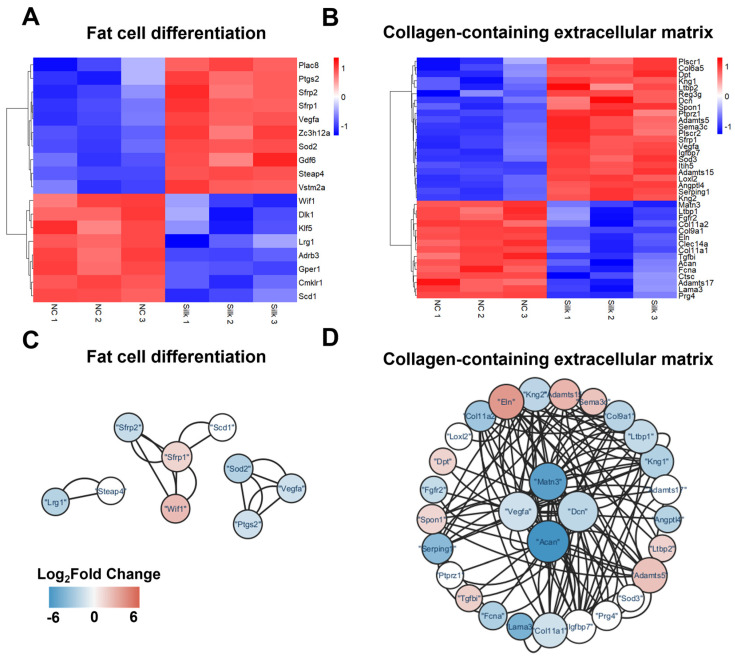
**Comparative analysis of mRNA expression between RSS-treated and control groups.** (**A**,**B**) Heatmaps displaying distinct expression patterns of DEGs associated with adipocyte differentiation and collagen-containing extracellular matrix in RSS-treated micromass cultures. (**C**,**D**) Protein–protein interaction networks of DEGs involved in adipocytes differentiation pathway and collagen-containing extracellular matrix pathway. The networks were visualized using Cytoscape 3.10.4, where node size reflects the degree of connectivity for each DEG.

Comprehensive analysis revealed 938 differentially expressed genes (DEGs), with 420 downregulated and 518 upregulated genes in the RSS group compared with the controls. A volcano plot was used to display the DEGs in the RSS group (Figure 5B). Gene ontology (GO) analysis categorized the DEGs into three aspects: biological processes (BP), cellular components (CC), and molecular functions (MF). Compared to the control, RSS group exhibited upregulation of genes positively regulating chondrocytes differentiation, collagen synthesis, and cell proliferation at the biological process level. At the molecular function level, genes associated with chondrocytes differentiation such as collagen trimer and collagen-containing ECM were dramatically upregulated, as well as glycosaminoglycan binding, G protein-coupled receptor binding, cytokine receptor binding and cytokine activity in the RSS group showed an upregulation trend (Figure 5C). This analysis corroborated previous findings, indicating upregulation of ECM-receptor interaction and promotion of chondrogenesis in RSS group. Further, the analysis of collagen-containing ECM in RSS group indicated that the four factors with the highest correlation were VEGFa, Matn, Dcn and Acan, which were closely related to collagen synthesis. The results showed that *VEGFa* and *Dcn* genes were upregulated and promoted chondral collagen synthesis; however, *Matn* and *Acan* genes were downregulated, which are associated with decreased collagen synthesis (Figure 6B,D). Although *Acan* expression was downregulated in the RSS-treated group according to RNA-seq analysis, Alcian Blue staining revealed increased accumulation of GAGs. This apparent discrepancy may reflect temporal decoupling between transcriptional activity and matrix deposition, as previously reported in chondrocyte culture systems [44]. Additionally, upregulation of *Dcn* may contribute to overall GAG content [45]. These data highlighted the complexity of extracellular matrix regulation during chondrogenesis and implied the complexity of the regulation of chondrogenesis by RSS.

Additionally, our data suggested that RSS inhibited adipogenesis. To further explain this phenomenon, multiple transcription factors associated with adipocyte differentiation and interaction between these factors were examined (Figure 6A,C). Notably, Wif1, an inhibitor of Wnt signaling, was greatly down-regulated in chondrocytes micromass cultured on RSS and it was also the most relevant factor in the regulation of adipogenesis. As well-known, several signaling pathways such as canonical Wnt/-catenin, Hedgehog and transforming growth factor beta (TGF-β) 1 and 2, as well as Sirtuin 1 (Sirt1), have been involved in adipocyte differentiation [46]. It has shown that canonical Wnt signaling inhibits adipogenesis and promotes osteoblastogenesis during development [47]. Downregulation of *Wif* leads to a decrease in the inhibition of Wnt signaling, i.e., an increase in Wnt signaling, which inhibits adipocyte differentiation and maturation. Therefore, RSS decreased adipogenesis by downregulation of Wif1. These findings aligned with the histochemical staining results and indicate that RSS has an effect of inhibiting the formation of lipid droplets and adipocyte differentiation. RSS–chondrocytes provided a simulated cartilage growth microenvironment for chondrocytes, particularly by upregulating *VEGFa* and *Dcn* mediated collagen synthesis and downregulating *Wif1* mediated inhibiting adipogenesis, thereby enhancing chondrogenesis.

### 3.6. ECM Production by Chondrocytes in Subcutaneous Transplantation Model

**Figure 7:**
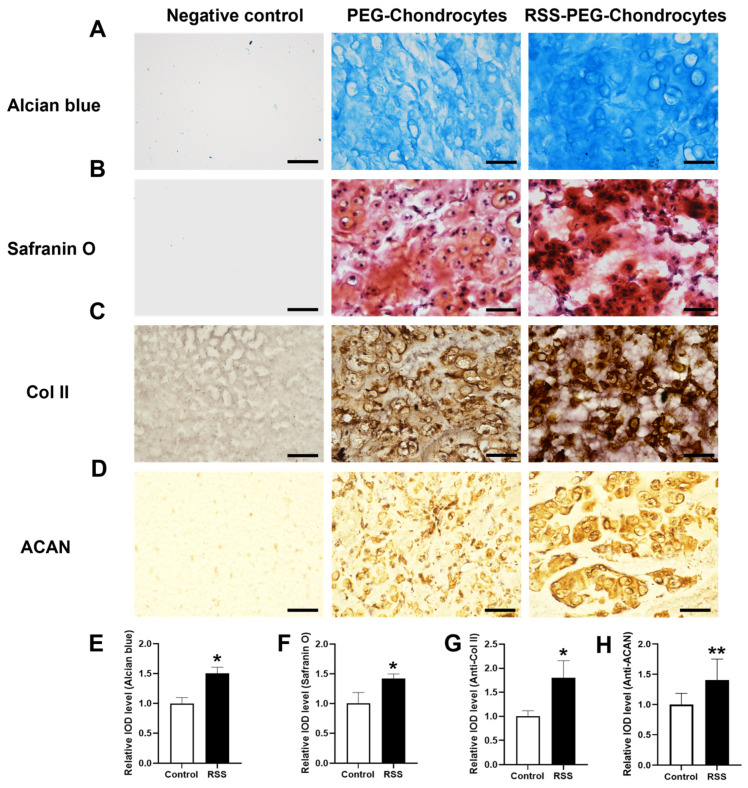
**RSS enhances engineered cartilage formation in the mouse subcutaneous transplantation model.** (**A**–**D**) Representative images of Alcian blue staining, Safranin O staining, and immunohistochemistry staining for Col II and ACAN. (**E**–**H**) Quantitative analysis of the relative IOD levels for each staining by Image J software 1.53. * *p* < 0.05, ** *p* < 0.01, *n* = 3. Scale bars: 50 μm.

The ECM components produced by chondrocytes, particularly GAG and Col II, are responsible for providing cartilage with its unique biomechanical properties [48]. The phenotypic maintenance of chondrocytes and the expression of cellular functions in the chondrocyte-biomaterial complex are critical for cartilage tissue engineering and the regeneration of neocartilage [49]. While the in vitro micromass culture system allows for detailed mechanistic investigation of RSS–chondrocyte interactions under controlled conditions, it does not fully recapitulate the complex physiological environment required for engineered cartilage maturation. In contrast, subcutaneous transplantation provides a vascularized three-dimensional host environment that facilitates nutrient diffusion, waste removal, and progressive tissue remodeling—processes that are critical for the formation of a well-organized extracellular matrix and the long-term maintenance of the chondrocyte phenotype. To assess whether RSS can enhance engineered cartilage formation within a more clinically relevant three-dimensional setting, a PEG-based hydrogel system was employed. This system offers a well-defined, biocompatible, and mechanically tunable matrix that supports chondrocyte encapsulation and extracellular matrix deposition, while enabling uniform incorporation of RSS and controlled implantation and retrieval. The PEG hydrogel used in this study was prepared according to a previously established formulation [17], which exhibited a compressive strength of approximately 20 MPa and a Young’s modulus of approximately 6 kPa. Given the relatively low RSS-to-PEG ratio, approximately 1:20 *w*/*w*, in the composite hydrogels, the bulk mechanical properties are expected to remain largely governed by the PEG network. However, direct mechanical characterization of the RSS–PEG composites was not performed in this study and warrants further investigation in future work. To analyze various ECM components, histochemical staining methods were employed, including Alcian blue staining to detect glycosaminoglycans, Safranin O staining to assess proteoglycan content, and immunohistochemical staining for Col II and ACAN to define the cartilage phenotype, as shown in Figure 7A–D. The integrated optical density for each staining was measured by image analysis and summarized in Figure 7E–H.

The staining results clearly demonstrate that chondrocytes in the RSS–PEG–Chondrocytes group maintained a spherical morphology, in contrast to the spindle-like morphology typically observed in two-dimensional cell cultures. The spherical shape, along with lacunae formation, is a characteristic feature of mature chondrocyte phenotype in cartilage [50]. The preservation of these phenotypic features enables the production of ECM, facilitating the formation of neocartilage.

Compared to the PEG–Chondrocytes group, a consistent increase in the relative IOD levels for each staining method was observed in sections of newly formed engineered cartilage tissue, indicating that RSS supports the maintenance of chondrocyte phenotypes and enhances ECM production. This, in turn, aids in the formation of neocartilage when co-incubated with RSS. Furthermore, whereas the PEG–Chondrocytes group exhibited discrete, island-like stained areas, the staining texture in the RSS–PEG–Chondrocytes group evolved into a centralized and localized network. This pattern reflects the close aggregation of chondrocytes, and this packed structure positively influences ECM secretion by reducing individual cell exposure to environmental oxygen. This mechanism enhances the chondrogenic capacity of chondrocytes and helps prevent dedifferentiation within the material-cell complex. Furthermore, the staining patterns observed in the RSS–PEG–chondrocyte group suggest that the incorporation of RSS into PEG hydrogels establishes a composite microenvironment conducive to cartilage regeneration and the preservation of chondrocyte phenotype. This effect may be attributed to the synergistic interaction between the chondrogenic properties of spidroin and the high hydration capacity and diffusion characteristics of PEG-based hydrogels. Such material–material interactions underscore the significant potential of RSS for application in hydrogel composite engineering. While the subcutaneous model successfully demonstrated that RSS enhances cartilage formation, it does not replicate the complex biomechanical and physiological environment of an articular joint. Therefore, future studies will employ an osteochondral defect model to evaluate RSS–PEG constructs under weight-bearing conditions. This model is essential for assessing scaffold performance under compressive and shear stresses, which critically influence chondrocyte behavior, extracellular matrix organization, and tissue integration with host bone and cartilage. Such evaluation is a necessary step toward determining the clinical potential of RSS–PEG composites for functional cartilage regeneration. In addition, while our study demonstrates enhanced GAG and collagen type II deposition in RSS-containing constructs, we acknowledge that Alcian Blue staining alone does not distinguish specific GAG subtypes. Safranin O staining, which interacts preferentially with chondroitin sulfate and keratan sulfate, and immunohistochemical staining for ACAN provided supportive evidence of cartilage-like proteoglycan accumulation in vivo. More detailed characterization of cartilage ECM components including chondroitin sulfate and keratan sulfate deserve to be further investigated. This represents an important direction for validating the cartilage-specific ECM components in the engineered cartilage formation using RSS as a bioscaffold.

## 4. Conclusions

Spider silk has emerged as a promising biomaterial for cartilage tissue engineering due to its exceptional mechanical properties, biocompatibility, tunable biodegradation rate, and capacity to support cell adhesion, proliferation, and differentiation [51]. However, the impracticality of harvesting native silk from spiders—a labor-intensive process yielding minimal quantities—has necessitated the development of alternative production strategies. To address this limitation and enable scalable manufacturing with enhanced consistency and quality control, synthetic biology approaches have been increasingly employed to produce recombinant spidroins in heterologous systems [52]. In the present study, we successfully engineered a novel protein-based RSS platform through molecular cloning, biomimetic spinning, and acid-induced coagulation. Co-incubation of RSS with murine chondrocytes in micromass culture demonstrated that RSS promotes chondrogenic differentiation, suppresses adipogenic commitment, and enhances cellular aggregation. These observations were corroborated by real-time PCR analysis, which revealed upregulation of chondrogenesis-related genes and downregulation of adipogenesis-associated markers. Moreover, RNA sequencing uncovered that RSS modulates the expression of genes involved in cartilage ECM remodeling. In vivo validation using a subcutaneous implantation model further confirmed that RSS enhances the deposition of chondrocyte-specific ECM components, including GAG and Col II. From a translational perspective, RSS offers significant advantages in terms of scalability and manufacturability. The recombinant production of RSS in *E. coli* enables high-yield fermentation with consistent quality, overcoming the limitations associated with harvesting native spider silk. In addition, the biomimetic spinning process relies on standardized and readily adaptable components, facilitating scale-up toward good manufacturing practice (GMP)-compatible production. In terms of immunogenicity, the use of SCID mice in this study was intentional to evaluate the intrinsic effects of RSS on chondrocyte behavior and ECM formation without confounding adaptive immune responses, a well-established approach for early-stage biomaterial validation [53]. However, this approach is appropriate for early-stage mechanistic studies, and we recognize that immunogenicity remains a critical consideration for any protein-based biomaterial intended for clinical use. RSS offers several advantages over native silk, including controlled composition, absence of sericin and gland-derived contaminants, reduced batch variability, and compatibility with stringent purification processes that minimize endotoxins and host cell proteins. These features are expected to reduce immunogenic risk. Nevertheless, as highlighted by Long et al. [54], silk-based biomaterials can exhibit antigenicity under certain conditions, and immune responses—though rare—may include allergic reactions or tissue necrosis. To address this, our RSS platform’s genetically programmable nature allows for further engineering to minimize immune recognition, such as epitope masking or incorporation of immunomodulatory domains. Furthermore, future studies will systematically evaluate RSS–PEG constructs in the immunocompetent animal models to assess long-term graft survival, immune compatibility and inflammatory responses including histological assessment of CD3+, CD4+, and CD8+ T-cell infiltration, macrophage polarization (M1/M2), and serum antibody responses (IgG, IgE). Such studies are essential prerequisites for clinical translation and will guide further material optimization. With a view toward clinical translation, future work will focus on optimizing the mechanical properties of RSS-based constructs to more closely mimic those of native cartilage, evaluating their performance in weight-bearing osteochondral defect models in larger animal systems, and systematically investigating long-term safety, physiochemical properties, degradation profiles, and regulatory considerations associated with recombinant protein-based biomaterials. In addition, the development of three-dimensional RSS-based scaffolds using extrusion-based bioprinting represents a promising direction, enabling further exploration of cell–material interactions and biological functionality within a physiologically relevant three-dimensional microenvironment. In summary, our study underscores the potential of RSS to preserve the chondrocyte phenotype, support the development of engineered cartilage tissue formation, and position RSS as a robust, scalable, and reliable biomaterial for future applications in cartilage tissue engineering and regenerative medicine.

## Figures and Tables

**Table 1:** Sequences of primers used for quantitative real-time PCR.

Gene	Forward Primer	Reverse Primer
*Sox9*	AGGAAGCTGGCAGACCAGTA	TCCACGAAGGGTCTCTTCTC
*Col2α* *1*	AAGGAGTTTCATCTGGCCCT	AGCAGGTCCTTGGAAACCTT
*Co* *l1α1*	ACATGTTCAGCTTTGTGGACC	TAGGCCATTGTGTATGCAGC
*Runx2*	CTTCACAAATCCTCCCCAAG	GAATGCGCCCTAAATCACTG
*Co* *l10α1*	CCTGGTTCATGGGATGTTTT	ACCAGGAATGCCTTGTTCTC
*PPARγ*	GGAAGACCACTCGCATTCCTT	GTAATCAGCAACCATTGGGTCA
*β-actin*	CCCAGAGCAAGAGAGG	GTCCAGACGCAGGATG

## Data Availability

The data presented in this study are available upon request from the corresponding author.

## Abbreviations

Sox9	SRY box transcription factor 9
Runx2	Runt related transcription factor 2
Col II	Collagen type II
Col X	Collagen type X
Col I	Collagen type I
PPARγ	Peroxisome proliferator-activated receptor gamma
ACAN	Aggrecan
RSS	Recombinant spider silk
ECM	Extracellular matrix
SEM	Scanning electron microscopy
EDX	energy dispersive X-ray spectroscopy
